# Polaron Delocalization and Transport in Doped Graphene Nanoribbon Thin Films

**DOI:** 10.1021/acsnano.5c03888

**Published:** 2025-07-07

**Authors:** M. Alejandra Hermosilla-Palacios, Sebastian Lindenthal, Justin D. Earley, Taylor J. Aubry, David DeLuca, Hashim Al Khunaizi, Alexander M. Spokoyny, Jana Zaumseil, Andrew J. Ferguson, Jeffrey L. Blackburn

**Affiliations:** † Materials, Chemical and Computational Science Directorate, 53405 National Renewable Energy Laboratory, Golden, Colorado 80401, United States; ‡ Institute for Physical Chemistry, 9144 Heidelberg University, 69120 Heidelberg, Germany; § Department of Chemistry and Biochemistry, 8783 University of California, Los Angeles, Los Angeles, California 90095, United States

**Keywords:** graphene nanoribbons, molecular doping, polarons, charge transport, carrier delocalization

## Abstract

Graphene nanoribbons (GNRs) are quantum-confined π-conjugated monolayer semiconductors with attractive properties for optoelectronic applications. However, the ground- and excited-state properties of charge carriers in GNRs are still poorly understood, particularly with regards to the coupling between charges and the GNR lattice and the degree to which this coupling impacts local and macroscopic charge transport. To address this issue, we systematically correlate carrier density-dependent charge transport with spectroscopic modulations in chemically doped thin films of armchair graphene nanoribbons (9-aGNRs). This study combines Fourier transform infrared (FTIR) and ultraviolet–visible–near-infrared (UV–vis–NIR) spectroscopy with both local and macroscopic conductivity measurements to arrive at a full and self-consistent picture of transport in doped GNR thin films. Using three different molecular p-type dopants (i.e., oxidants), we demonstrate that hole polarons are the dominant quasi-particle determining charge transport in GNRs and that the degree of polaron delocalization depends sensitively on the dopant and the hole density. For all three dopants, the local conductivity probed by microwave spectroscopy substantially exceeds the long-range conductivity obtained by four-point probe measurements. Interestingly, the dopant size substantially influences charge transport at high hole densities. We ascribe this effect to different propensities for forming bipolarons with lower mobilities than polarons. Comparison of GNR transport and spectral properties to other prototypical π-conjugated semiconductors (e.g., semiconducting polymers or carbon nanotubes) benchmark the charge transport properties of GNR thin films for optoelectronic devices and applications.

## Graphical abstract

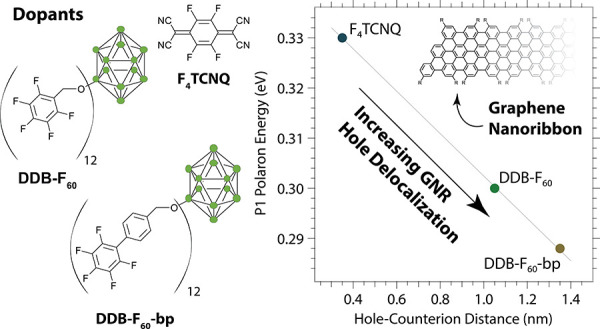

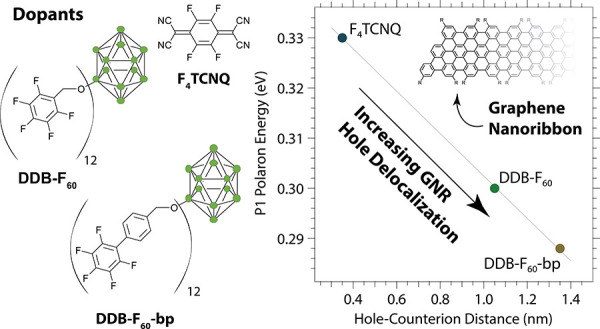

## Introduction

Solution-processed semiconductors can enable a range of low-cost, flexible, and multifunctional optoelectronic devices and applications. Key examples include energy harvesting devices such as solar cells and thermoelectric devices;^1–3^ spintronic and quantum information devices;^4^ transistors and digital/analog logic elements;^5,6^ and multifunctional wearables and sensors. Electronic doping, the intentional manipulation of atomic/molecular constituents or local fields to tune carrier density and Fermi level, is a fundamental approach for optimizing semiconductors and their heterojunctions for such optoelectronic devices.^7,8^ Detailed studies on doped solution-processed semiconductors in the past two decades have elucidated fundamental carrier density-dependent transport mechanisms that can guide rational device development.^7,9^

Graphene nanoribbons (GNRs) are single sheets of graphene with a narrow width (<20 nm) that provides lateral quantum confinement, width-dependent optical and electrical bandgaps, and potentially exotic properties arising from e.g., spin-dependent edge states.^10–12^ Opening a bandgap in the typically semimetallic graphene density of states in laterally confined nanoribbons^11–13^ makes GNRs interesting for electronic devices (e.g., digital logic) that build on tailor-made optical properties. Previous studies have demonstrated or suggested large binding energies (around 700 meV), Frenkel-like character, and strong dipole anisotropy of excitons in GNRs.^14–16^ Some experimental reports have also suggested strong vibronic coupling in the GNR optical response, which contrasts with how GNRs are often modeled with purely electronic transitions.^16^ Advances in solution-based synthesis^17–20^ have made GNRs with precise geometries accessible on a large scale and hence enable their application as solution-processable semiconductors. Prior theoretical and experimental research on doping of GNRs has focused on the incorporation of substitutional atoms (e.g., B, N) and functional group edge modification.^21–23^ However, it is challenging to achieve fine-tuning of the carrier concentration and Fermi level with these doping strategies especially on a large scale. Much less attention has been paid to molecular redox dopants for carrier density modulation in GNRs. This strategy has been particularly successful for tunable doping of molecular and polymeric semiconductors,^24,25^ single-walled carbon nanotubes (SWCNTs),^2,25^ mono- to few-layered transition metal dichalcogenides (TMDCs),^26^ and metal halide perovskites.^8^ Despite numerous theoretical studies,^28–30^ there are only limited experimental data on the extent to which charge carriers in GNRs couple to dopant counterions and/or the graphene lattice and the impact of this coupling on charge carrier transport.

The cross-correlation of spectroscopic and electrical transport measurements has been a successful approach to further the understanding of charge transport in solution-processed semiconductor thin films.^31–35^ Varying degrees of electron–hole and charge carrier–lattice interactions in these materials can produce different quasi-particles with unique spectral and transport signatures. The typical excited states produced by photon absorption in these low-dielectric semiconductors are excitons,^36^ Coulomb-bound electron–hole pairs, and the sharp excitonic optical transitions are strongly attenuated (bleached) by the presence of excess (majority) charge carriers.^33,34^ The same ground-state charges can produce a variety of characteristic spectral signatures depending on the nature of the semiconductor, including broad free carrier absorption in the near- to far-infrared,^37,38^ polaronic optical transitions arising from carriers bound to local lattice distortions,^29,39^ and trion optical transitions arising from carriers bound to excitons.^34^ Additionally, strong absorption coefficients for charges in the gigahertz and terahertz frequency regions allow for informative microwave^34^ and THz^20,40^ conductivity measurements. The strong dependence of the optical response of charges on carrier density and (de)­localization in the above-mentioned spectroscopic measurements has provided crucial mechanistic insights into similar carrier density-dependent electrical transport measurements in systems like semiconducting polymers^31,35^ and SWCNTs.^32–34^

Although numerous spectroscopic and electrical transport studies exist for GNRs, most were performed on isolated individual GNRs on substrates. Such samples are amenable to local spectroscopies (e.g., Raman and scanning tunneling spectroscopies)^18,41–43^ and transport measurements focusing on short transport distances between lithographically patterned electrodes or aligned GNR field-effect transistors.^44,45^ Furthermore, the photoconductivity of GNR dispersions and thin films has been studied via optical-pump terahertz-probe spectroscopy.^46,47^ Much less common are complementary spectroscopic and transport studies on GNR thin films that focus on macroscopic charge transport, and even fewer studies exist for redox-doped GNRs with fine-tuned carrier density.^39^ In this study, we systematically cross-correlate optical spectroscopy and electrical transport in solution-processed 9-aGNR thin films that are doped with different molecular redox dopants to achieve finely tuned carrier (hole) densities. We find that the optical spectra and transport properties of redox-doped 9-aGNR thin films can be described self-consistently by a framework that considers hole polarons as the dominant charge carriers in the p-type graphene nanoribbons. The conductivity (both local and long-range) and polaron transition energies depend in complex ways on both the dopant identity and carrier density, effects that we ascribe to the balance between hole–counterion Coulomb attraction in polarons and the dopant-dependent propensity to form bipolarons at high carrier density. Our results provide support for existing theories on the role of polarons and bipolarons in transport within GNRs and identify potential strategies to improve electronic transport within doped GNR networks.

## Results and Discussion

### Optical Properties of Doped Graphene Nanoribbon Thin Films

Graphene nanoribbons (GNRs) obtained from conventional solution-based reactions often present poor stability and processability.^48^ In this study we employ a previously developed synthesis method^19^ for 9-aGNRs with branched alkyl side chains (for molecular structure see Figure 1 **A, top**) that make them reasonably stable in toluene and tetrahydrofuran (THF) especially after further separation by liquid cascade centrifugation (LCC).^38,39^ The synthesized 9-aGNRs were subjected to a low rotational centrifugal force (<200 g) to achieve stable dispersions of GNRs that contain less defects than dispersions subjected to higher centrifugal forces.^38^ Dispersions and thin films of 9-aGNRs are chemically doped by three different dopants: 2,3,5,6-tetrafluoro-tetracyanoquinodimethane (F_4_TCNQ), an aryl-functionalized dodecaborane cluster with 60 fluorine atoms (DDB-F_60_), and a similar DDB cluster with an additional aryl group between the DDB cluster and the fluorine-modified aryl group (DDB-F_60_-bp) (see Figure 1A, bottom and Figure S1). These dopants differ with respect to the van der Waals (vdW) distance between the charge located at the ‘geometrical center’ of the counterion^50^ and the carrier injected into an organic semiconductor host, which we estimate varies from 0.35 nm for planar F_4_TCNQ that can π-stack with the GNRs to >1 nm for the sterically bulky functionalized DDB dopants (Figure 1A), which in turn is expected to impact the electron–hole Coulomb interaction.^32,35,51,52^ Geometric parameters of the molecules are based on prior crystallographic data,^50^ and Density Functional Theory (DFT) calculations indicate that electrons are delocalized over the boron cluster,^52^ justifying the use of the geometrical center of the DDB counterions. Based on the Coulomb interaction between an electron located on the dopant counterion and a hole located on the GNRs, increased counterion size and thus electron–hole separation translates to a weaker Coulomb attraction (binding energy) and enhanced hole delocalization.

**Figure 1:**
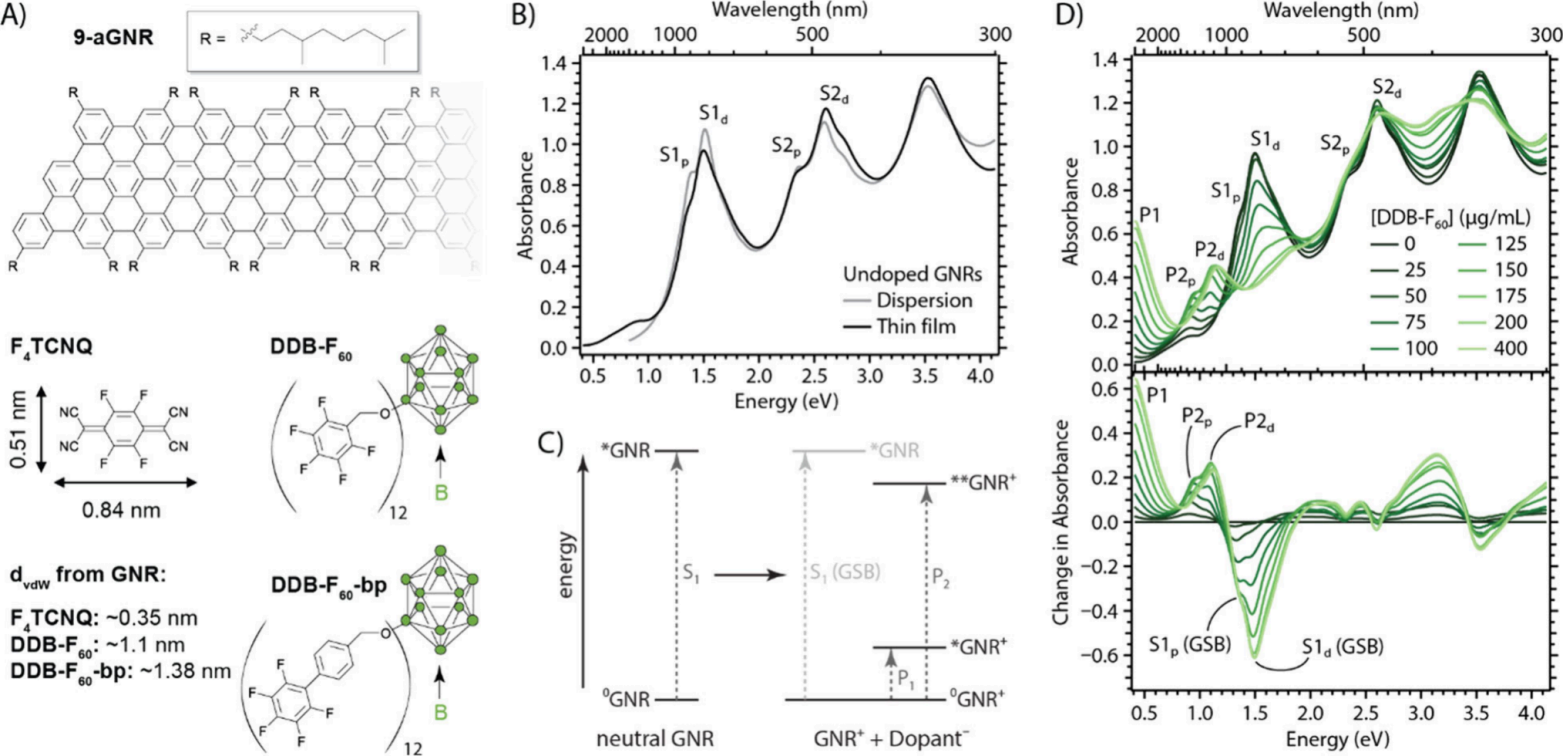
(A) Chemical structures of 9-armchair graphene nanoribbons (9-aGNRs), the three different dopant molecules: F_4_TCNQ; DDB-F_60_; DDB-F_60_-bp. The structures for DDB dopants only show one fluorinated substituent for easier visualization, but the dopants are fully and symmetrically functionalized on all boron vertices. Refer to Figure S1 for full structure of DDB-F_60_. The estimated van der Waals distance (d_vdW_) between anion center-of-mass and the doped GNRs is also provided. (B) Absorption spectra of undoped 9-aGNR dispersion (in toluene) and thin film. The lowest-energy exciton transitions for pristine and defective 9-aGNRs are labeled as S1_p_ and S1_d_, respectively. (C) Schematic Jablonski diagram illustrating optical transitions of a neutral GNR to form an exciton (left) and associated with polarons in a doped GNR illustrating a weakening (bleach) of the ground-state absorbance of the neutral GNR (right). (D) Steady-state absorbance (top) and differential absorbance (bottom) for DDB-F_60_-doped 9-aGNR thin film with increasing dopant concentration. The lowest-energy exciton ground-state bleach (GSB) and highest-energy polaron (P2) transitions for pristine and defective 9-aGNRs are labeled as S1_p_ (GSB)/P2_p_ and S1_d_ (GSB)/P2_d_, respectively. The lowest-energy polaron (P1) transition is also labeled without distinction of the pristine and defective components.

9-aGNR films were prepared by an ultrasonic spray coating technique that has been used to prepare high-quality films of single-walled carbon nanotubes (SWCNTs)^53,54^ and semiconducting polymers.^55^ Dispersions and films made from 9-aGNR dispersions show a series of well-defined absorption bands between 300 and 1000 nm (Figure 1B). The lowest energy transition envelope is dominated by a strong peak at 840 nm (1.476 eV) and a shoulder at 920 nm (1.348 eV). Lindenthal et al. used time-domain density functional theory in combination with Raman, absorption and photoluminescence spectroscopy to assign these to two different 9-aGNR species (pristine and defective),^38^ here labeled as S1_d_ (for defective) and S1_p_ (for pristine). A similar two-peak envelope in the range of 400–600 nm can be observed for the second excitonic transition (S2) of the pristine and defective 9-aGNRs. The defects were identified as unclosed carbon–carbon bonds at the edges, resulting from incomplete cyclodehydrogenation during organic synthesis. This also rationalizes the assignment of the higher energy peak to the defective species, as the disruption in the GNR lattice leads to a stronger confinement of the exciton wave function and thus higher energy transitions.

Figure 1C shows a schematic Jablonski diagram used to interpret the changes to the absorbance spectrum due to chemical doping of the 9-aGNRs films. The Jablonski diagram can be applied to either the pristine or defective species. In our experimental data we have both species present, and each will have the three different transitions depicted in the right side (GNR^+^ + Dopant^–^) diagram so we differentiate them with the subscript p for pristine and d for defective. P-type doping extracts electron density, reducing the oscillator strength of the lowest energy exciton transition (S_1_), producing a so-called ground-state bleach (GSB) whose intensity grows with increasing carrier density (Figure 1D). In π-conjugated polymers, structural and electronic modifications in the polymer backbone in the vicinity of a charge^56–59^ produce new ‘polaronic’ electronic levels above and below the highest-occupied molecular orbital (HOMO) and lowest-occupied molecular orbital (LUMO) levels (valence and conduction bands), respectively, that result in low-energy optical transitions.^31^ Polarons are quasi-particles where charge carriers and phonons couple via local lattice distortions around the charge carrier.^56–59^ Lindenthal et al.^38^ determined that the doping-induced optical absorbance features observed between 1000 and 1400 nm for F_4_TCNQ-doped 9-aGNRs in solution also correspond to polarons,^60,61^ suggesting the mechanistic picture in Figure 1C may adequately describe doped GNR thin films as well. However, lower energy transitions (e.g., P1) could not be observed in the relatively narrow spectral window of that study.

Figure 1D shows UV–vis–NIR spectra obtained for a single 9-aGNR thin film subjected to a series of DDB-F_60_ dopant solutions with progressively increasing concentration, and Figure 1E shows differential doping spectra, obtained by subtracting the undoped spectrum from each spectrum taken for a particular dopant concentration. Figure S2 plots similar steady-state and differential spectra for F_4_TCNQ and DDB-F_60_-bp-doped GNR thin films. Increasing p-doping results in the expected GSB of the S1_d_ and S1_p_ exciton transitions (Figure 1C).^34,38^ It also leads to the emergence of several new red-shifted absorption bands. Based on the conventional nomenclature from the polymer community (Figure 1C)^38^ we assign these transitions to the P2_d_ (1.11 eV) and P2_p_ (0.91 eV) polaron transitions for the defective and pristine GNRs respectively (Figure 1E). This assignment is based on the sequential emergence of the two features upon increasing doping level: The P2_p_ transition emerges at low doping levels upon a bleach of the S1_p_ transition, while the P2_d_ only is visible for higher doping levels, when the S1_d_ transition is bleached (Figure 1E). This behavior has also been observed in our recent study on doping of 9-aGNRs in dispersion, in which we additionally corroborated this assignment by theoretical DFT calculations.^38^ The additional strong and low-energy doping-induced absorbance feature is consistent with the so-called P1 polaron transition, although the full shape of this feature (needed to confirm the P1 assignment) cannot be resolved in this spectral range using UV–vis–NIR absorbance spectroscopy.

To confirm that this low-energy feature observed in the UV–vis spectra of Figure 1E is indeed characteristic of a polaron transition, we paired UV–vis–NIR and FTIR spectroscopy of 9-aGNR films deposited onto KBr single crystals. Figure 2A displays representative spectra of 9-aGNR thin films doped to approximately the same doping level (based on the extent of the S1 GSB) with the three different p-type dopants. Each doped spectrum shows a symmetric Voigt line shape = peaking around 0.3 eV, consistent with the expectation of a polaronic transition. The symmetric line shape of a polaron peak contrasts with the so-called ‘free carrier absorbance’ response of mobile charge carriers that are not strongly bound as polarons to lattice distortions. For free carriers the infrared absorbance increases monotonically with decreasing photon energy, typically with a power law behavior. Unlike the P2 transition envelope, we cannot discern two distinct transitions within the P1 transition envelope that can be assigned to pristine and defective GNRs. The absorbance changes observed in Figures 1 and 2 suggest that the traditional polaronic framework and nomenclature from the polymer community (Figure 1C) can be successfully used to describe charge carriers in chemically doped GNRs.

**Figure 2:**
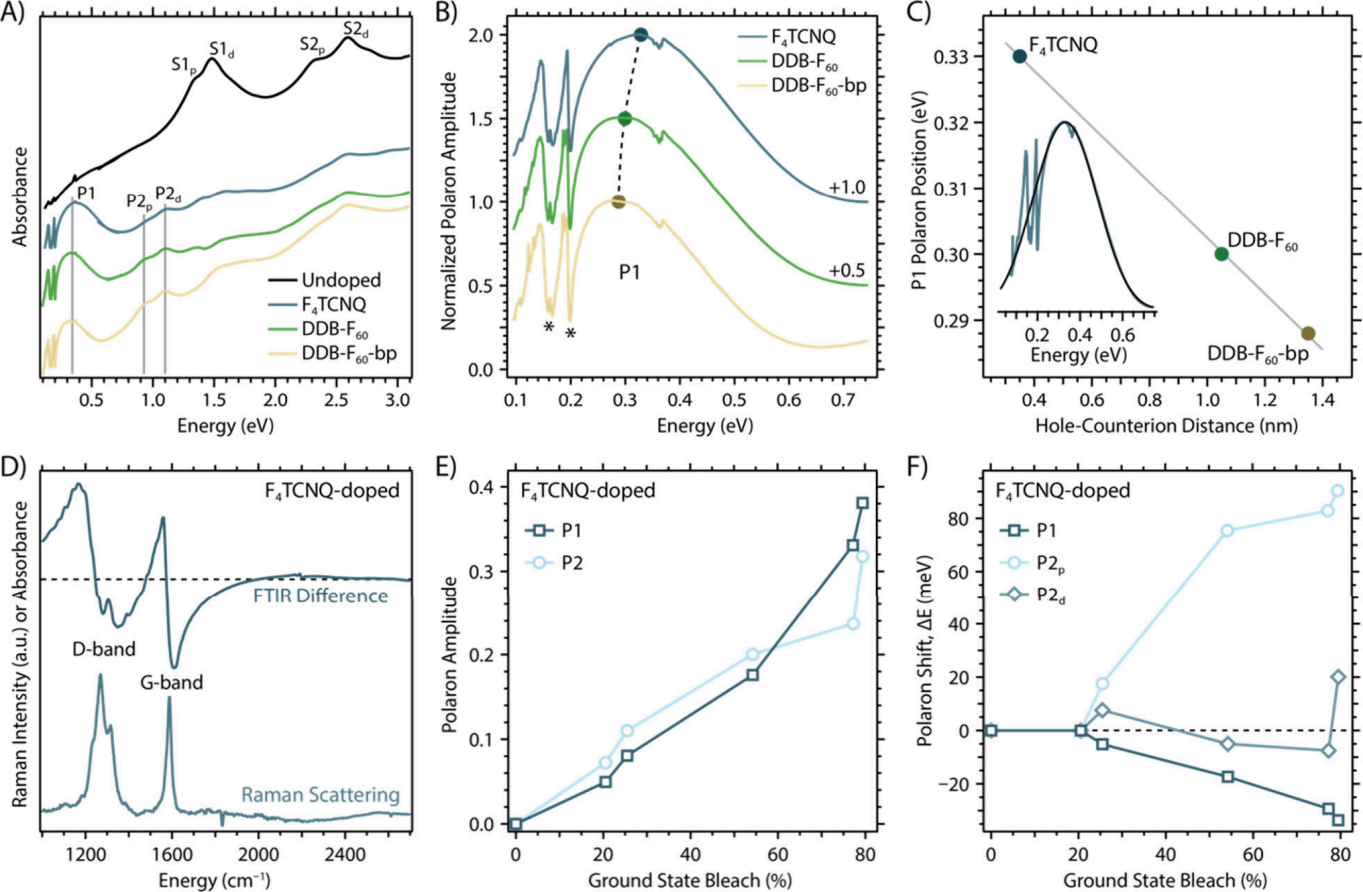
(A) Paired UV–vis–NIR and FTIR spectra for 9-aGNR films deposited on KBr, both undoped and doped with the three p-type dopants to similar doping levels. The first and second excitonic transitions for the pristine (S1_p_ and S2_p_) and defective (S1_d_ and S2_d_) 9-aGNRs are identified together with the corresponding polaron transitions (P1, P2_p_ and P2_d_). (B) Zoomed-in spectra of the P1 transitions for the spectra shown in panel A. These spectra are generated by subtracting the undoped spectrum from the doped spectra to remove scatter associated with the KBr substrates, and are normalized to the peak of the P1 absorbance. (C) Correlation of the P1 polaron peak energy to the distance between the hole on the GNR and the dopant counterion, showing a linear trend. Inset: Representative Voigt fit of the P1 peak for the F_4_TCNQ-doped 9-aGNR film, used to obtain accurate P1 energies and FTIR difference spectra. (D) Comparison of the FTIR difference spectra, obtained via Voigt fitting (e.g., panel C inset) of F_4_TCNQ-doped 9-aGNR film, compared to the Raman spectrum of 9-aGNRs (785 nm excitation). The sharp resonances in the difference spectrum align with the Raman modes corresponding to defect (D) and in-plane vibrational modes (G). (E) P1 and P2 (P2_p_ and P2_d_ combined) amplitudes as a function of the ground state bleach (S1_p_ and S1_d_ combined) for F_4_TCNQ-doped 9-aGNR film. (F) Polaron peak energies, obtained via multipeak fitting for F_4_TCNQ-doped 9-aGNR film, as a function of ground state bleach.

The polaron framework developed for semiconducting polymers allows for useful analyses of the nature of charge carriers in 9-aGNRs. For example, Aubry and co-workers showed for a doped polymer^51^ that the P1 polaron peak shifts to lower energies as polarons become more delocalized. Figure 2B presents the zoomed-in P1 spectra of 9-aGNR films doped with three different dopants. The P1 peak shifts to lower energy in the sequence of F_4_TCNQ to DDB-F_60_ to DDB-F_60_-bp. Figure 2C plots the polaron P1 peak energy position, obtained via a Voigt fit (inset of Figure 2C), as a function of the hole–counterion distance (Figure 1A) for the dopants. The fitted P1 peak energy indeed shifts to lower energies as the hole–counterion distance increases, suggesting that the 9-aGNR polaron delocalization is correlated with this distance due to lower Coulomb interaction, i.e. a more delocalized polaron.

A second interesting observation is the presence of sharp resonant features, marked with asterisks in Figure 2B, that are superimposed on the broad P1 peaks in the range of ca. 0.1–0.2 eV. Figure 2D plots the difference spectrum obtained for the F_4_TCNQ-doped film by subtracting the Voigt fit of the P1 peak (Figure 2C) from the experimental P1 spectrum. We find good agreement between the peaks observed in this difference spectrum and the Raman modes observed for undoped 9-aGNRs, with a peak envelope centered at 1300 cm^–1^ comprised of the “defect band” (D) and C–H vibrations and a 1600 cm^–1^ peak corresponding to the in-plane vibration (G band). These peaks are consistent with infrared-active vibrational or Fano-like resonances that have been observed in SWCNTs,^37^ graphene,^62,63^ and conducting polymers,^64,65^ and arise from the coupling of electronic transitions of polarons or free carriers to discrete vibrational modes of the π-conjugated semiconductor.

Additional analysis of the polaron’s amplitude and the change in polaron peak energy (Δ*E*) is shown in Figure 2E and F, respectively, using the F_4_TCNQ-doped 9-aGNR film as an example. As the doping concentration increases (higher ground-state bleaching) the polaron amplitudes increase (Figure 2E), which is consistent with a transfer of oscillator strength from excitonic transitions to the polaron transitions (Figure 1C). The polaron peak energies (Figure 2F) show distinct behaviors when considering P1, P2_p_, and P2_d_ separately. P1 shifts to lower energies (negative Δ*E*) as the doping concentration increases (higher ground-state bleaching). As discussed above, the lowest energy polaron transition shifts to lower energies with increasing delocalization of charges in doped polymers.^51^ The continuous red-shift of P1 indicates a higher degree of carrier delocalization in the F_4_TCNQ-doped 9-aGNR film with increasing hole density. This density-dependent delocalization is consistent with the overlap of dopant-localized potential wells along the GNR’s backbone, an effect also observed in doping series of SWCNTs^66,67^ and inferred from carrier density-dependent thermoelectric transport measurements in conducting polymers.^68^

In contrast to the P1 polaron energy, the P2_p_ transition shifts toward higher energy. This behavior for P2_p_ can be explained by Figure 1C, which shows that a decreased P1 transition energy should be offset by an increase in the P2 transition energy. It is not immediately clear why P2_p_ would show this shift toward higher energies while P2_d_ remains mostly unchanged. We speculate that the polarons on defective GNRs may become localized at the defect sites, preventing the degree of delocalization that can be achieved in pristine GNRs. However, it is also important to point out that the P1 transition is likely a convolution of two polaronic transitions, arising from the defective and pristine GNRs, that we cannot reliably deconvolute without additional separation of these two distinct GNR populations.

Importantly, the detailed analysis shown in Figure 2E,F requires a multipeak fitting routine that includes the contribution of dopant counterion spectra that arise in the doped 9-aGNR films (see Supporting Information, Figure S3). The spectra are particularly complex in the visible and NIR regions where spectral components of the P2 9-aGNR polaron, S1 9-aGNR excitonic transitions, and dopant anion peaks can overlap with each other. In contrast, the P1 peak is relatively spectrally isolated, has a simple Voigt line shape, and its intensity should relate directly to the hole density in the GNRs at a particular doping level (Figure 2E). As such, we use the area of the P1 peak, normalized by the thickness of a given film, as a proxy for the volumetric hole density in the forthcoming charge transport analyses.

### Transport of Polarons and Bipolarons

Charge transport measurements were performed with a linear four-point (4-pt) probe setup and in a dark microwave (ca. 10 GHz) conductivity apparatus (refer to methods). Figure 3A–C shows the progressive growth of hole density in the 9-aGNR films (reflected by the thickness-normalized P1 area, P_1_ A/t), 4-pt probe conductivity, and microwave conductivity as the concentration of each dopant is increased. The concentration range used in these experiments corresponds to the range over which no obvious dopant aggregation, due to solubility limits, was observed. Within each separate doping series, increasing the dopant concentration increases the conductance measured by both methods and the P1 polaron area. These clear trends corroborate that each p-type dopant leads to hole-doping of the 9-aGNRs that, despite coupling of the charges to local lattice distortions as polarons, can contribute to both local (microwave) and long-range (4-pt) conductivity. Consistent with prior studies on semiconducting SWCNTs and polymers, the hole density and resulting conductivity can be systematically tuned by the concentration of the dopant solution in which the 9-aGNR thin film is soaked.^32,33,51^ For all samples at all carrier concentrations, the microwave conductivity exceeds the 4-pt probe conductivity by a factor of ca. 4–6, a trend we discuss in more detail below.

**Figure 3:**
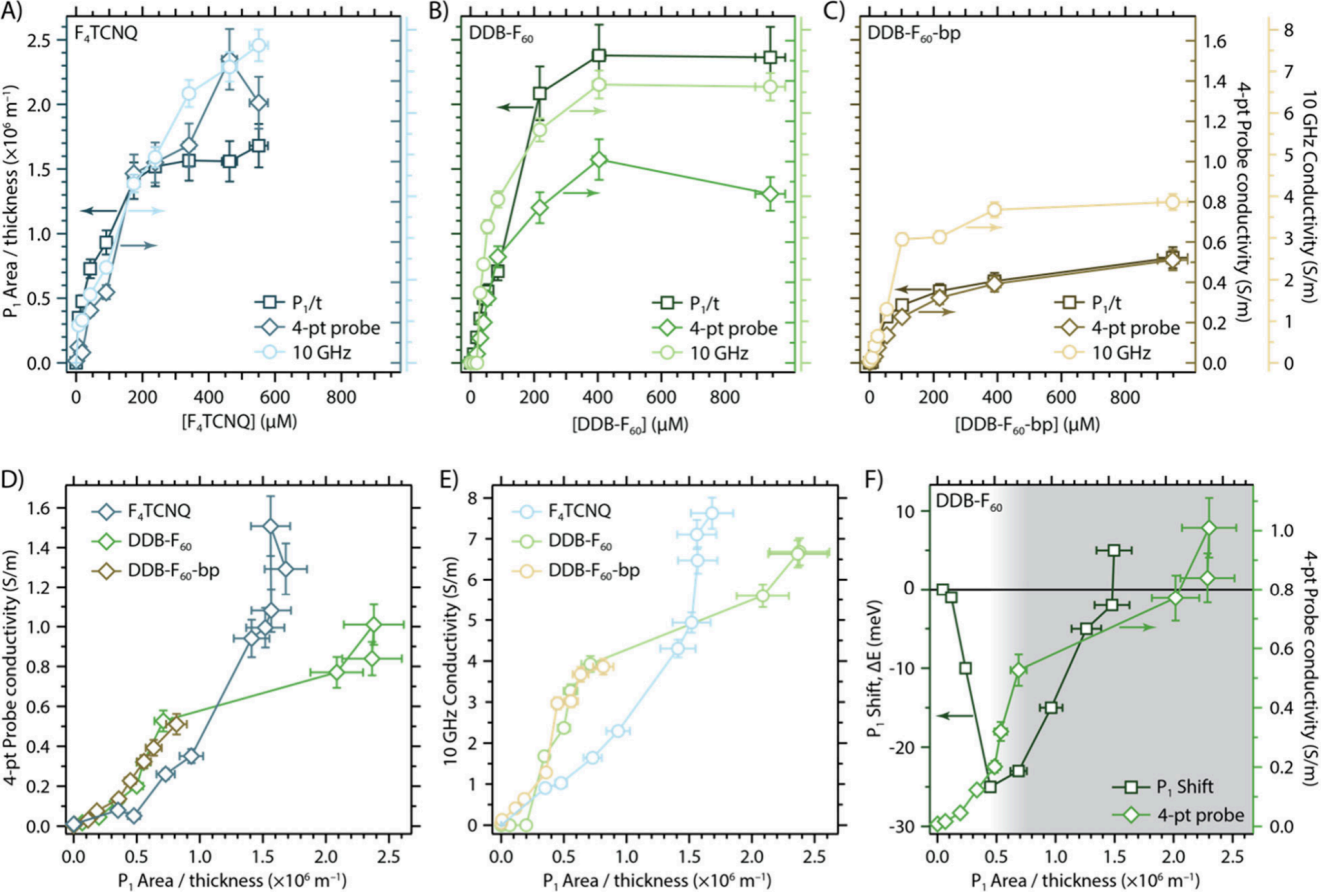
(A–C) Thickness-normalized P1 polaron area (squares), four-point probe conductivity (diamonds) and microwave conductivity (circles) for doped 9-aGNRs as a function of dopant concentration for (A) F_4_TCNQ, (B) DDB-F_60_, and (C) DDB-F_60_-bp. (D) Four-point probe conductivity and (E) microwave conductivity at ca. 10 GHz for 9-aGNRs doped by all three dopants as a function of thickness-normalized P1 polaron area. (F) Energy shift (Δ*E*) of P1 polaron peak (left axis) and 4-Point probe conductivity (right axis; from panel B) as a function of thickness-normalized P1 area for a representative DDB-F_60_-doped 9-aGNR film. The transition between noninteracting polaron transport and transport impacted by interacting polarons and/or bipolarons is indicated by the gradient between the unshaded region at low hole densities to the gray shaded region at moderate to high hole densities.

Figure 3D,E compares the carrier density-dependent 4-pt probe (Figure 3D) and microwave (Figure 3E) conductivity for 9-aGNR films doped with the three different dopants. These plots reveal two interesting regions of conductivity across these doping series. In the low to moderate range of conductivities (ca. 0.1–0.5 S m^–1^ for 4-pt probe and ca. 1.5–4 S m^–1^ for microwave), the DDB-doped films tend to have higher conductivity values than the F_4_TCNQ-doped film. The very low conductivity values deviate somewhat from this trend, but these conductance values are near the limits of detection for these measurements, so they have a relatively large degree of uncertainty. The higher conductivity in DDB-doped 9-aGNRs films could be due to the larger distance between the DDB-F_60_ counterion and the hole on the GNR, in comparison to the F_4_TCNQ hole–counterion distance. A larger hole–counterion distance is expected to increase the hole mobility due to decreased Coulomb interaction and binding energy. These trends qualitatively match the trends in P1 peak energy, which we propose correlates inversely with polaron delocalization, as discussed above (Figure 2B,C).

Interestingly, above 4-pt probe and microwave conductivity values of ca. 0.5 S m^–1^ and 4 S m^–1^, respectively, the DDB-F_60_ doped 9-aGNR film conductivities plateau, while the conductivity values for the F_4_TCNQ-doped film continue to grow with increasing hole density. Returning to the hypothesis that the P1 polaron peak energy correlates with polaron delocalization, we plotted the carrier density-dependent P1 energy shift in Figure 3F. Unlike the case for F_4_TCNQ-doped 9-aGNR films, where the P1 peak energy continuously shifts to lower energy with increasing carrier density (Figure 2F), the P1 peak energy first shifts to lower energy (P_1_ A/t = 0–0.5 × 10^6^ m^–1^) and then back to higher energy as doping density increases (above P_1_ A/t = 0.5 × 10^6^ m^–1^). We note that the P1 peak energy data were analyzed for a different DDB-F_60_ doped 9-aGNR film than the one used for the analyses in the other panels, so the extent of doping may be subtly different between the two samples. Despite these differences, plotting the trends of 4-pt probe conductivity and P1 polaron peak shift on the same plot suggests a clear correlation between the two trends. Conductivity increases rapidly over the first ca. 30% of the injected hole density (i.e., up to the gradient between the unshaded and gray shaded regions in Figure 3F), while the P1 peak rapidly red-shifts over the same hole density (P_1_ A/t = 0–0.5 × 10^6^ m^–1^). This steep slope of conductivity then plateaus in the same range of hole density at which the P1 peak begins to blue-shift (above P_1_ A/t = 0.5 × 10^6^ m^–1^). Both metrics even show a similar abrupt change (sharp decrease in conductivity and increase in P1 peak energy) at the highest attained carrier densities. These trends are reproducible across multiple different samples.

We hypothesize that the correlation in conductivity and P1 peak energy for DDB-F_60_ doped 9-aGNR films reflects a carrier density-dependent transition across different (but not necessarily discrete) regimes of transport: transport dominated by noninteracting polarons at low carrier densities (unshaded region of Figure 3F) and transport impacted by the contribution of polaron–polaron interactions and/or bipolarons at moderate to high carrier densities (gray shaded region of Figure 3F). Bipolarons are quasiparticles consisting of two bound polarons and typically form only at carrier densities where interaction between polarons becomes significant. Bipolarons have been predicted for semiconducting polymers and graphene nanoribbons,^29,69,70^ and a recent theoretical report even studied their properties specifically in 9-aGNRs.^69^ Two key properties of bipolarons motivate our hypothesis. First, bipolarons are thermodynamically stabilized with respect to polarons, and thus have a smaller single-particle bandgap^69^ (equivalent to the P2 energy in Figure 1C). This smaller bandgap places the singly occupied molecular orbital (SOMO) in Figure 1C farther away from the GNR valence band (or HOMO level), translating to a higher P1 transition energy, as is observed for DDB-F_60_ doped GNR in the gray region of Figure 3F. Second, relative to a polaron, the higher charge density and associated stronger lattice deformation increases the bipolaron’s effective mass and decreases its mobility, as is suggested by the reduction in the slope of conductivity in the gray region of Figure 3F. Cassiano et al. estimate that the bipolaron mobility is more than an order of magnitude lower than that of a polaron in a representative cove-type GNR (4-aGNR).^29^

It is important to note that the conductivity is a product of the carrier density and mobility, and that the carrier mobility likely has a complex, nonmonotonic relationship to the carrier density, for both polarons and bipolarons. For instance, we expect the polaron mobility to initially increase with carrier density due to reduction in depth of the polaron potential well, due to some combination of a dopant-induced increase in the local dielectric constant,^71^ potential well overlap that reduces the effective barrier to charge transport,^32^ and/or filling of trap states. As the dopant and carrier density increases, the distribution of conducting quasiparticles should gradually transition from a distribution dominated by polarons (low density) to a mixed distribution (moderate density) to a distribution dominated by bipolarons (high density). The total conductivity is the sum of the conductivities for the polaron and bipolaron populations.

The plateau in conductivity for DDB-F_60_ doped 9-aGNR films at moderate carrier densities is an interesting contrast to the apparent absence of such a plateau in conductivity for F_4_TCNQ-doped 9-aGNR films at similar carrier densities. We speculate that this contrast may arise from the more delocalized nature of polarons in DDB-F_60_ doped 9-aGNRs, due to the larger counterion separation, relative to F_4_TCNQ. Perhaps nonintuitively, more delocalized polarons should have a higher probability of interacting at a lower hole density than more localized polarons. Thus, this plateau region may reflect a regime in which a combination of polaron–polaron interactions (e.g., scattering) and/or bipolaron formation can contribute to mobility and conductivity reduction in the DDB-F_60_ doped 9-aGNRs. The decrease of conductivity observed at the highest dopant concentrations for both DDB-F_60_ and F_4_TCNQ-doped GNRs is similar to the behavior commonly attributed to bipolaron-*dominated* transport in doped polymers.^72^ We suggest that the complex density-dependent coupling of holes to their molecular dopant counterions, other hole polarons, and to the GNR-lattice deserves further experimental and theoretical consideration.

### Comparing Polaron and Free Carrier Transport in Organic Semiconductors

To better contextualize the transport and spectroscopy studies of the doped 9-aGNR thin films, we present a comparison to two other quintessential π-conjugated semiconductor thin film systems – semiconducting polymers (here poly­(3-hexylthiophene), P3HT) and semiconducting single-walled carbon nanotubes (s-SWCNTs). First, the comparison of the microwave conductivity to that measured by 4-point probe can provide valuable information on transport within a given thin-film semiconductor. Microwave conductivity probes diffusive transport occurring on the time scale of the 10 GHz probe cycle time of ca. 100 ps. Such short time scales imply that the microwave measurement probes transport at a much more local scale than the 4-pt probe measurement, which requires transport over length scales of millimeters or longer. Figure 4A shows a correlation between the 10 GHz conductivity and 4-point probe conductivity where the gray diagonal line is the 1:1 relationship. Independently of the dopant, all the 9-aGNRs data sets are above the diagonal line, barring one spurious data point, reflecting a higher local conductivity relative to the long-range transport. This GNR behavior contrasts sharply with that of the semiconducting polymer P3HT and small-diameter s-SWCNTs, in which we observe that the local conductivity generally matches the long-range conductivity (values along the diagonal).

**Figure 4:**
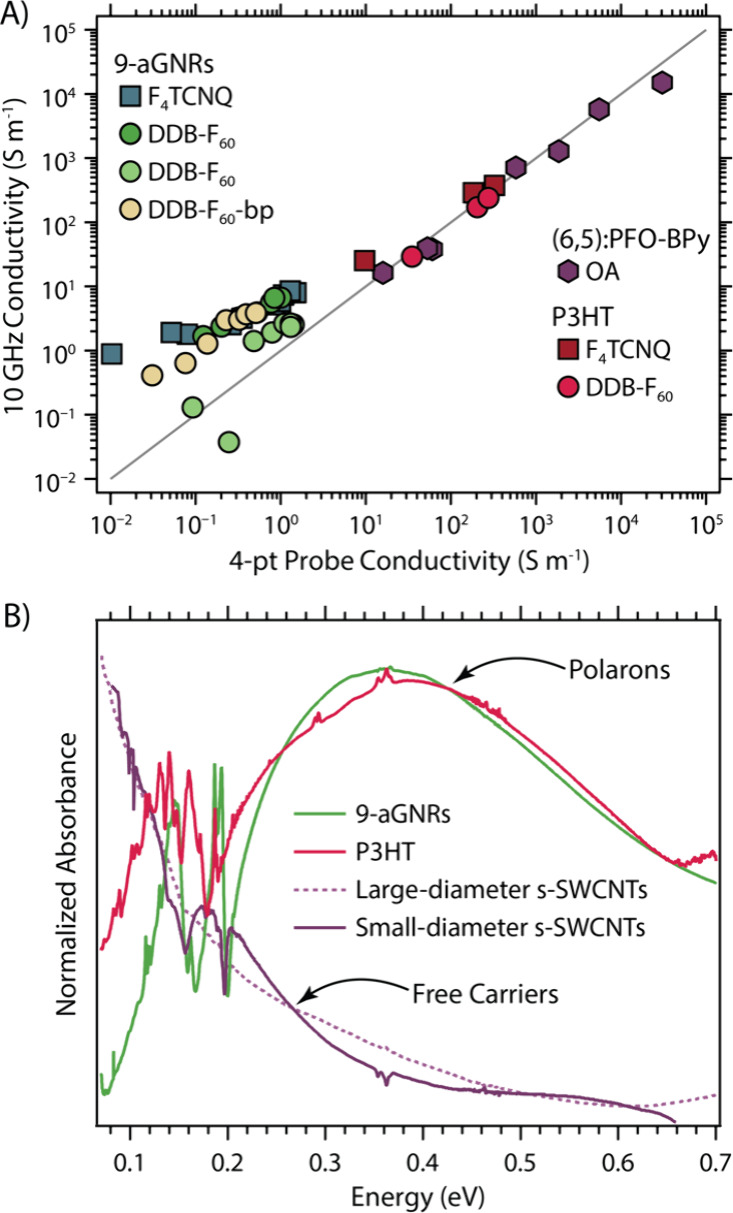
(A) Comparison of the 10 GHz microwave (local) versus 4-pt probe (long-range) electrical transport behavior of small-diameter s-SWCNTs doped by triethyloxonium hexachloroantimonate (OA); P3HT polymer doped by F_4_TCNQ or DDB-F_60_; and 9-aGNRs doped by F_4_TCNQ, DDB-F_60_, DDB-F_60_-bp. (B) Free carrier absorption for OA-doped large- and small-diameter s-SWCNTs, compared to the strong polaron absorption features for DDB-F_60_-doped 9-aGNRs and P3HT.

Returning to the insights that FTIR spectra can provide on the nature of carriers in these doped semiconductors, Figure 4B displays the FTIR spectra of heavily doped 9-aGNRs, P3HT, and two different (large- and small-diameter) s-SWCNT thin films. The spectra for doped s-SWCNTs show an absorption that grows monotonically with decreasing energy, characteristic of a so-called “free carrier absorption band” observed for conventional semiconductors like silicon.^73^ This free carrier band contrasts sharply with the strikingly similar polaronic transitions observed for doped 9-aGNR and P3HT films. The observation that polarons dominate the transport in both 9-aGNRs and P3HT suggest that the different local and long-range conductivity behavior observed for 9-aGNR thin films in Figure 4A cannot be attributed solely to the polaronic nature of the carriers. Instead, it must arise from an intrinsic property of the 9-aGNRs, or their thin films, that inhibits long-range conductivity to some degree.

We speculate that morphological properties of the solution-processed GNR thin films may play a role in diminishing the long-range conductivity. The lengths of individual 9-aGNRs (15–25 nm) are much shorter compared to s-SWCNTs (hundreds of nm to tens of μm) and conjugated polymers (low hundreds of nm for reasonable molecular weights, e.g. >50 kDa). It is expected that the short length and peripheral substitution of the 9-aGNRs leads to a morphology dominated by discrete domains of π-stacked GNRs with limited electronic overlap or connectivity between neighboring GNR aggregates. This contrasts with SWCNTs films, where the individual nanotubes form a well-connected mesh,^53,74,75^ and in high-quality semiconducting polymer thin films that feature interconnected networks of large crystalline aggregates.^76–78^ Thus, we suggest two strategies to improve the long-range conductivity of GNR thin films: (1) synthesis of GNRs with longer average lengths and (2) development of thin-film deposition methods that can tailor and optimize aggregate alignment and the interconnection between aggregates within a film.

## Conclusion

In this work we studied charge transport in doped films of 9-aGNRs and correlated both microscopic and macroscopic conductivity with spectral features. As previously suggested^38^ charge-induced, red-shifted absorption features are of polaronic origin very similar to π-conjugated polymers and in contrast to s-SWCNTs. We find a clear correlation of the dopant size (i.e., anion distance from GNR) with both decreasing P1 polaron energy and increasing carrier mobility for larger dopants, as observed previously for polymers^51^ and s-SWCNTs.^32^ Data obtained at high doping levels hint toward the formation of bipolarons in the doped 9-aGNRs, a phenomenon which has previously only been predicted for graphene nanoribbons.^69,70^

Importantly, the correlation of spectroscopic and conductivity measurements establishes a direct connection between polaronic signatures and an increase in conductivity, suggesting a polaron-assisted charge transport mechanism, as predicted by several theoretical studies.^28,79–81^ These mechanistic insights align with the prediction of exceptionally high electron–phonon interactions in GNRs as compared to more rigid nanomaterials such as SWCNTs^81,82^ and points to similarities in charge transport in polymers and solution-synthesized GNRs. Hence, strategies developed for the improvement of polymer-based electronics could be applicable to enhancing device performance of GNR thin films. The discrepancy between local and long-range conductivities of 9-aGNR films at the same doping levels (i.e., charge carrier densities) in contrast to model polymers and s-SWCNTs further highlight the need for longer GNRs and improved film formation techniques for more ordered domains (e.g., aligned ribbons) and higher interconnectivity.

## Methods

### 9-aGNR Synthesis and Processing

Atomically precise 9-aGNRs with a length of 15 to 25 nm were synthesized according to an adapted protocol by Li et al.^19^ The exact synthesis route was reported previously.^38^ In a sealed 25 mL round-bottom flask, a mixture of 9-aGNR powder in THF or toluene (1 mg mL^–1^) was ultrasonicated for 4 h in a Branson 2510 sonication bath while the temperature was kept constant at room temperature. To sediment the pristine 9-aGNRs, the dispersion was centrifuged for 1.5 h at 200 g in a Hettich Mikro 220R centrifuge, equipped with a 11.95A fixed-angle rotor. The supernatant was discarded and the sediment redispersed in fresh solvent.

### 9-aGNR Film Deposition

9-aGNR thin films were prepared through ultrasonic spray deposition^53^ using a dispersion flow rate of 0.3 mL/min and gas flow rate of 7.0 std L/min. The nozzle power was fixed at 0.8 W, and the substrate was heated to 130 °C to allow for evaporation of the solvent. After spraying the films, they were soaked in a hot toluene bath (80 °C).

### Molecular Doping

9-aGNR thin films were p-doped by soaking films in solutions of a particular dopant with varying dopant concentrations at room temperature. In some cases, films were also “de-doped” to achieve a desired lower hole concentration. Dedoping was achieved by soaking the GNR film in a solvent that the dopant was soluble in, either at room temperature or at slightly elevated temperatures, depending on the desired extent of dedoping.

### Absorption Measurements

UV–vis-NIR absorption measurements were carried out on a Varian Cary 7000 spectrometer utilizing an integrating sphere or on a Varian Cary 5000 spectrometer in transmission mode without an integrating sphere. Fourier transform infrared spectroscopy (FTIR) measurements were carried out on thin films that were spray-coated onto KBr single crystal substrates. FTIR measurements were performed on a Thermo-Nicolet 7600 FTIR spectrometer in transmission mode.

### Raman Spectroscopy

Raman spectra of drop-cast GNR dispersions were recorded on a Renishaw inVia confocal Raman microscope equipped with a 50× long working distance objective (N.A. 0.5, Olympus) at an excitation wavelength of 785 nm. To minimize the influence of spot-to-spot variations, we averaged over spectra of >200 spots. The spectra are baseline-corrected to account for the PL background.

### Dark Microwave Measurements

Dark microwave conductivity measurements were performed in an X-band microwave cavity, as described previously.^83,84^ For each doping condition, the microwave resonance of the samples was measured at least three times, with the microwave cavity deconstructed and the sample rotated by 180° between each resonance measurement (while keeping the film oriented toward the end of the cavity defined by the circular iris). To calculate the conductance of each sample, we use the commercially available COMSOL Multiphysics (v 6.0) finite element package to solve Maxwell’s equations for the electromagnetic field distribution within the cavity and obtain simulated resonance curves for a wide range of sample conductance. Each simulated resonance curve is fitted using a Lorentzian function, and the Lorentzian fit parameters are tabulated as a function of sample conductance, generating a lookup table that is subsequently used to fit the experimental data and obtain sample conductance.

### 4-Point Probe Sheet Resistance Measurements

DC conductivity of the GNRs films was measured with a Lucas Signatone Corporation linear 4-point probe sheet resistance setup with the SP4–40045OBY probe head (Osmium probes with ca. 250 μm probe tip radius and a 1 mm probe spacing), as described previously.^85–87^ Current–voltage curves are generated by sourcing current between the two outer probe tips from a Keithley 2400 Source-Measure Unit and measuring the potential difference between the two inner probes. For each doping condition, the samples were measured seven times in different areas of the ca. 1 cm × 2 cm rectangular sample area for different relative orientations between the linear probe tips and the long axis of the substrate, and the values were averaged.

## Supplementary Material



## Notes

Structures of F_4_TCNQ and DDB-F_60_ obtained from crystallography data; Steady-state and differential absorbance spectra for F_4_TCNQ and DDB-F_60_ doped GNR thin films; Example of multipeak fitting of absorbance spectra for doped GNR thin film; Modeling microwave conductivity data to extract 9 GHz conductivity (PDF)

## Abbreviations

GNRs: graphene nanoribbons (9a armchair)
LCC: liquid cascade centrifugation
THF: tetrahydrofuran
F_4_TCNQ: 2,3,5,6-tetrafluoro-tetracyanoquinodimethane
DDB-F_60_: dodecaborane cluster with 60 fluorine atoms (bp biphenyl)
PL: photoluminescence
AFM: atomic force microscopy
SWCNTs: single-walled carbon nanotubes
OA: triethyloxonium hexachloroantimonate
P1: polaron 1
P2_d_: polaron 2 for defective GNRs
P2_p_: polaron 2 for pristine GNRs
DFT: Density Functional Theory
FTIR: Fourier transform infrared
GSB: ground state bleach
HOMO: highest-occupied molecular orbital
LUMO: lowest-occupied molecular orbital
P3HT: poly­(3-hexylthiophene)
SOMO: singly-occupied molecular orbital
S1: lowest energy exciton transition
S2: second exciton transition
TMDC: transition metal dichalcogenides
vdW: van der Waals
