# Supplementary material for: Polaron Delocalization and Transport in Doped Graphene Nanoribbon Thin Films
Source: ACS Nano. 2025 Jul 7;19(28):25732–43. doi: 10.1021/acsnano.5c03888 (PMC12291592; doi:10.1021/acsnano.5c03888)
Supplement: Supplementary file 1 [file nn5c03888_si_001.pdf]

Supporting Information: Polaron Delocalization and Transport in Doped Graphene Nanoribbon Thin Films

M. Alejandra Hermosilla-Palacios,[1] Sebastian Lindenthal,[2] Justin D. Earley,[1] Taylor J. Aubry,[1] David DeLuca,[3] Hashim Al Khunaizi,[3] Alexander M. Spokoyny,[3] Jana Zaumseil,[2] Andrew J. Ferguson,[1*] Jeffrey L. Blackburn[1*]*

[1] Materials, Chemical and Computational Science Directorate, National Renewable Energy Laboratory, Golden, Colorado 80401, United States

[2] Institute for Physical Chemistry, Heidelberg University 69120 Heidelberg, Germany

[3] Department of Chemistry and Biochemistry, University of California, Los Angeles, California 90095, USA

Section S1. Structures of F_4TCNQ and DDB-F_{60}

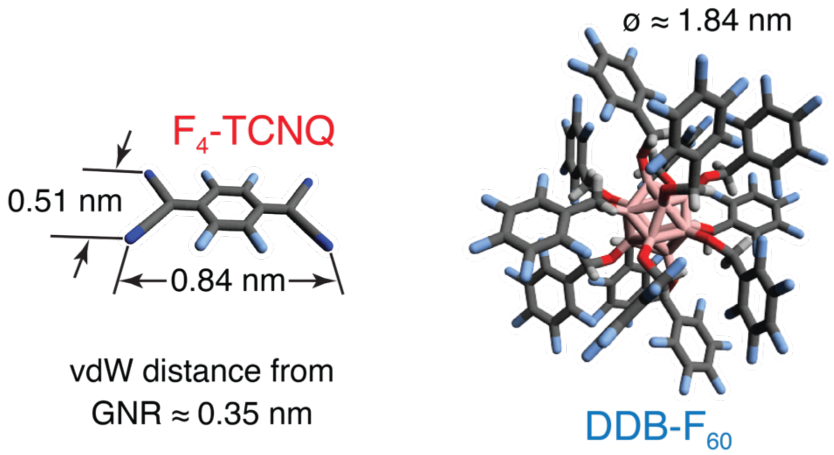

Figure S1. Dopant chemical structures obtained from crystallography data, where we considered geometric parameters to estimate the van der Waals distance from the GNR. Both DDB dopants are fully functionalized with fluorinated substituted phenyl groups making them symmetric.

Section S2. Steady-state and differential absorbance spectra for F_4TCNQ and DDB-F_{60} doped GNR thin films

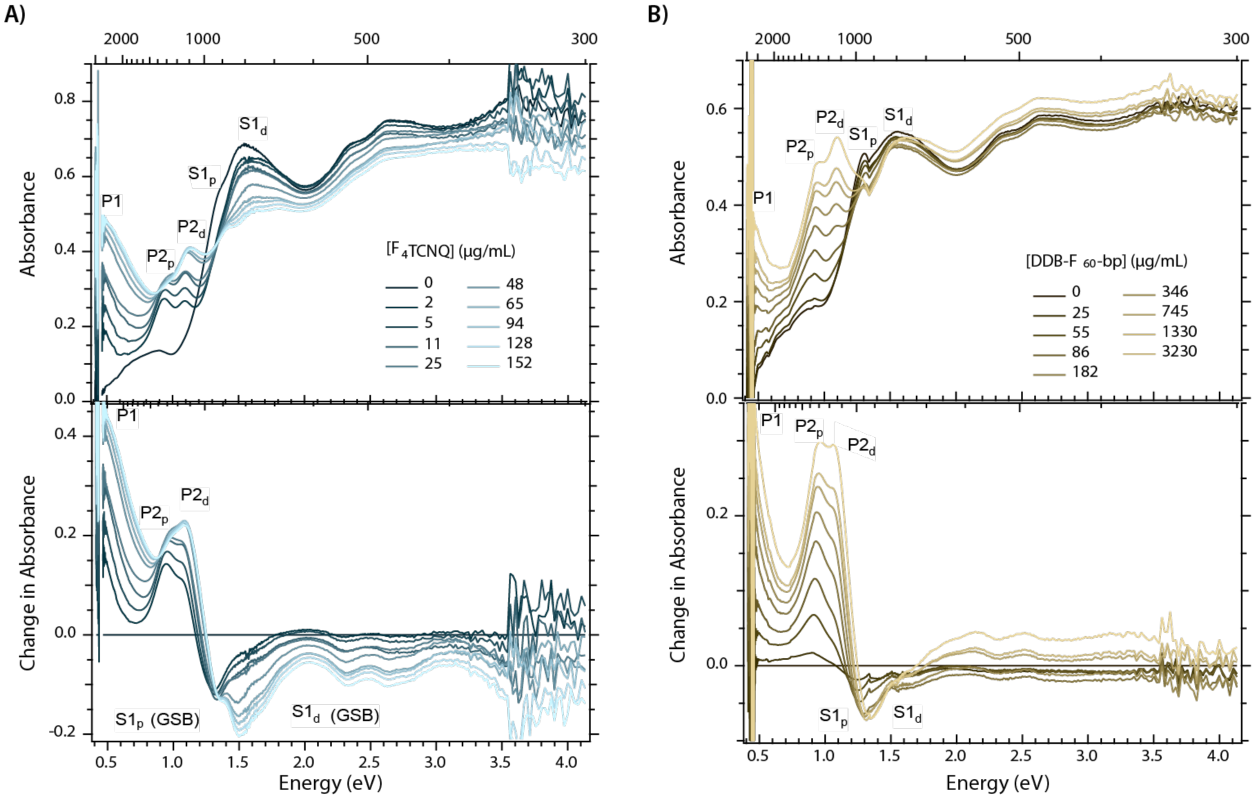

Figure S2. Steady-state absorbance (top) and differential absorbance (bottom) for **A)** F_4TCNQ and **B)** DDB-F_{60}-bp doped 9-aGNR thin films with increasing dopant concentration. The lowest-energy exciton ground-state bleach (GSB) and highest-energy polaron (P2) transitions for pristine and defective 9-aGNRs are labeled as $S1_p$ (GSB)/$P2_p$ and $S1_d$ (GSB)/$P2_d$, respectively. The lowest-energy polaron (P1) transition is also labeled without distinction of for pristine and defective 9-aGNRs.

Figure S2A (S2B) top panel shows a series of UV-Vis-NIR spectra obtained for a single 9-aGNR thin film subjected to a series of F_4TCNQ (DDB-F_{60}-bp) dopant solutions with progressively increasing concentration, and **Figure S2A (S2B)** bottom panel shows differential doping spectra, obtained by subtracting the undoped spectrum from each spectrum taken for a particular dopant concentration. Increasing p-doping results in the expected GSB of the $S1_d$ and $S1_p$ exciton transitions. It also leads to the emergence of several new red-shifted absorption bands. We assign these transitions to the $P2_d$ (1.11 eV) and $P2_p$ (0.91 eV) polaron transitions for the defective and pristine GNRs respectively. The additional strong and low-energy doping-induced absorbance feature is consistent with the so-called P1 polaron transition, although the full shape of this feature (needed to confirm the P1 assignment) cannot be resolved in this spectral range.

Section S3. Example of multi-peak fitting of absorbance spectra for doped GNR thin film.

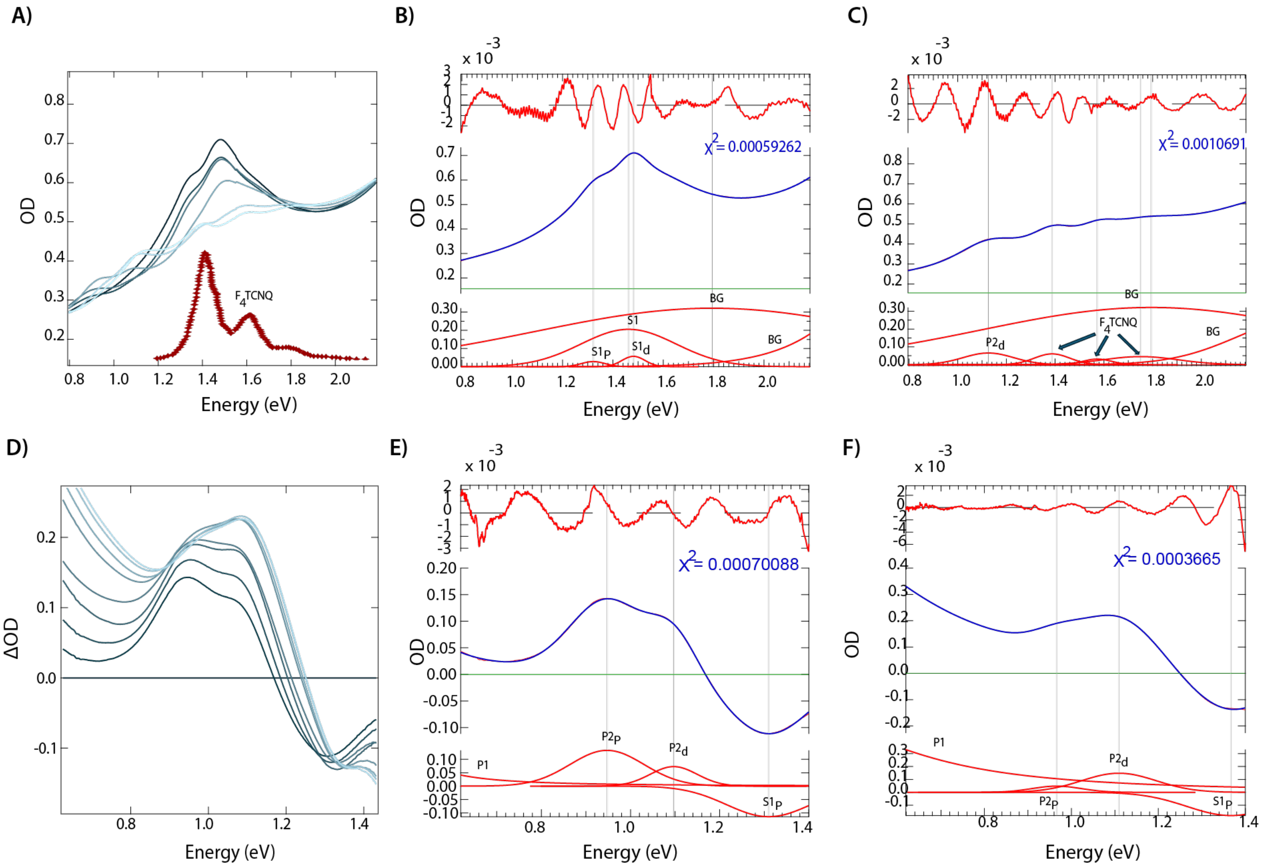

Figure S3. A) Steady-state absorbance of F_4TCNQ doped 9-aGNR thin film with increasing dopant concentration showing the region used to calculate the area under the curve for the GSB correcting for anion absorbance of the dopant (red trace). **B)** Neat 9-aGNR film absorption fitted to identify $S1_d$, $S1_p$ and high-energy background. **C)** Based on the identified excitonic transition, P2d and P2p can be fitted and fitted by keeping the high-energy background constant a good correction for the anion absorbance is obtained. **D)** Differential absorbance of F_4TCNQ doped 9-aGNR thin film with increasing dopant concentration showing the region used to calculate P1, $P2_d$ and $P2_p$ area under the curve. **E)** F_4TCNQ (2 µg/mL) doped 9-aGNR thin film fitting P1 (Voigt 0), $P2_d$ (Voigt 1), $P2_p$ (Voigt 2) and GSB (Voigt 3). **F)** F_4TCNQ (152 µg/mL) doped 9-aGNR thin film fitting P1 (Voigt 0), $P2_d$ (Voigt 1), $P2_p$ (Voigt 2) and GSB (Voigt 3).

Figures S3A-F show the different steps used to calculate and correct the area under the curve for the different transitions mentioned in the main manuscript. **Figure S3A** shows the F_4TCNQ doping progression and the dopant anion spectrum for the data shown in **Figure 3E** and **3F**. First, the absorbance of the neat film is fitted (**Fig. S3B**) to identify the electronic transitions ($S1_p$ and $S1_d$) and the background contribution (BG peaks). The high-energy background peaks are kept constants for all doped samples. For a given doped spectrum, we identify $P2_p$, $P2_d$ and the anion contribution based on the absorbance spectrum (F_4TCNQ peaks in **Fig. S3C**). The area under the curve for the anion is used to correct for GSB calculation based on the dopant concentration and cleanest anion fit. Next, the differential absorbance (**Fig. S3D**) is fitted to identify P1, $P2_p$, $P2_d$ and calculate the area under the curve based on the Voigt

profiles computed from the multi-peak fitting procedure in Igor. **Figure S3E** and **Figure S3F** show the fitting results for two different doping steps: 2 µg/mL and 152 µg/mL respectively. In these two figures the following peaks and areas are obtained: P1, $P2_p$, $P2_d$ and GSB (S1).

Section S4. Modeling microwave conductivity data to extract 9 GHz conductivity.

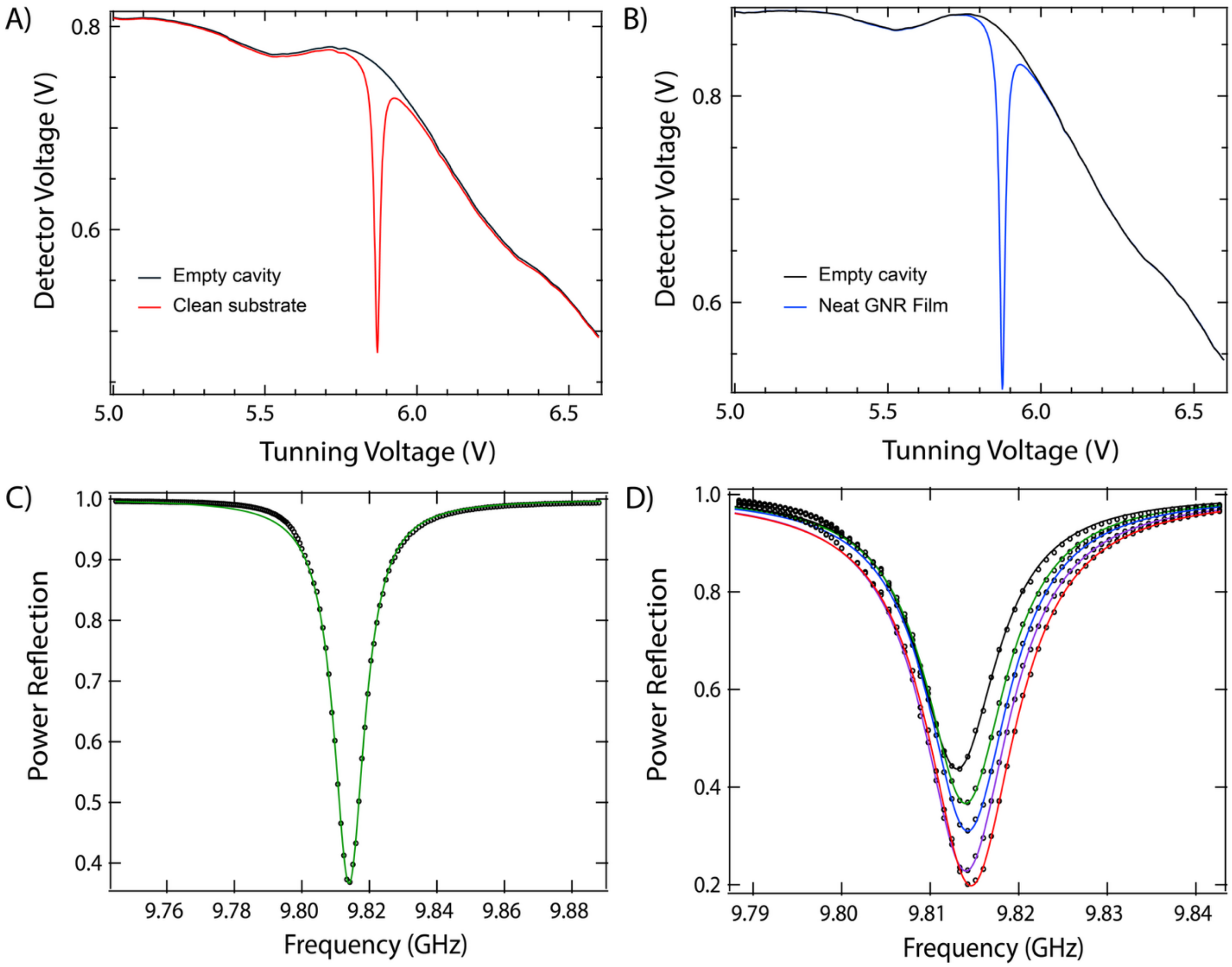

Figure S4. Measured detector voltage as a function of tunning voltage of F_4TCNQ doping series 9-aGNR thin film for the **A)** clean substrate (red curve) and its corresponding empty cavity response (black curve); and **B)** Neat 9-aGNR film (blue curve) and its corresponding empty cavity response (black curve). **C)** Calculated (green curve) and measured (black markers) resonance curves for the microwave conductivity after solving Maxwell‘s equations. **D)** A few of the doping steps and corresponding resonance curves obtained from the IGOR routine, the resonance curves deepen as the doping concentration increases.

Dark microwave conductivity measurements are performed using an optimized X-band resonant microwave cavity, modeled and custom designed using COMSOL Multiphysics (v 6.0) using the optional RF package. This software allows for solving Maxwell’s equations for propagation of electromagnetic radiation through microwave waveguide circuits and cavities. In the system employed here, we apply a voltage to the voltage-controlled oscillator (VCO, Sivers V03262X/00) as the source, producing 100 mW

of microwave power, which is tunable from 8.4-13.5 GHz, and record the microwave detector voltage (Figures S4A and S4B). The system is carefully calibrated to convert the source voltage to microwave frequency and the detector voltage to measured microwave power.

The experimental frequency-dependent power reflection coefficient from the loaded resonant microwave cavity (i.e., the cavity with a clean/blank substrate or the cavity with a substrate onto which the thin film is deposited), which we call the "resonance curve" (black markers in Figures S4C and S4D), is calculated via comparison to the response of the empty cavity. Experimental data for the empty and loaded cavity, as received from the IGOR data acquisition interface, is plotted in Figure S4A and S4B. To account for any drifts in the ambient temperature and changes in day-to-day operation, the response of the empty cavity is measured at multiple points before, during, and after measurements of the loaded cavity.

COMSOL Multiphysics (v 6.0) is also used to model the frequency-dependent power reflection coefficient from the loaded resonant microwave cavity as a function of the conductance of the sample generating a lookup table that is used to fit the experimentally measured resonance curve and obtain the sample conductance (solid lines in Figures S4C and S4D). The thin film conductance is corrected for the contribution to the measured resonance curve due to the clean/blank substrate and converted into the conductivity using the dimensions of the thin film.
